# Assessment of the risk factors for impending fractures following radiotherapy for long bone metastases using CT scan-based virtual simulation: a retrospective study

**DOI:** 10.1186/s13014-014-0227-1

**Published:** 2014-10-16

**Authors:** Zuzana Tatar, Martin Soubrier, Anne Françoise Dillies, Pierre Verrelle, Stéphane Boisgard, Michel Lapeyre

**Affiliations:** Medical Oncology Department, Centre Jean Perrin, Centre de Lutte contre le Cancer de la Région Auvergne, 58 Rue Montalembert, BP 392, Clermont-Ferrand, 63011 France; Rheumatology Department, CHU Gabriel Montpied, 58 Rue Montalembert, BP 69, Clermont-Ferrand, 63003 France; Radiotherapy Department, Centre Jean Perrin, Centre de Lutte contre le Cancer de la Région Auvergne, 58 Rue Montalembert, BP 392, Clermont-Ferrand, 63011 France; Orthopedic Surgery Department, CHU Gabriel Montpied, 58 Rue Montalembert, BP 69, Clermont-Ferrand, 63003 France; Département d’Oncologie Médicale, Hôpital de Jour, Centre Jean Perrin, Centre de Lutte contre le Cancer de la Région Auvergne, 58, rue Montalembert, B.P. 392, Clermont-Ferrand, 63011 France

**Keywords:** Long bone metastases, Fractures, Radiotherapy

## Abstract

**Background:**

Radiotherapy for long bone metastases (RTLB) can be complicated by fractures, which considerably increase morbidity and mortality. The aim of this study was to analyze the risk factors for impending fractures following radiotherapy for long bone metastases (RTLB) using CT scan-based virtual simulation.

**Methods:**

Forty-seven (47) patients were treated with RTLB (18 lung, 11 breast, 10 prostate and 8 other cancers) for a period of 18 months. Two doctors analyzed the CT images prior to radiation therapy. The impending fractures were then monitored and the correlation between bone scan parameters and fracture occurrence was analyzed.

**Results:**

The male gender ratio was 0.57 and the mean age 62.8 (33–93) years. The average size of the metastatic lesions was 32 (8–87) x 2 (6–81) x 52 (7–408) mm with cortical involvement (CI) in 66% of cases. The site was in the upper third of the bone in 92% of cases (28 femoral, 17 humeral and two tibial).

Ten fractures occurred: two during RTLB, seven after one month and one after 6.6 months. The fractured lesions measured 48 (17–87) x 34 (12–66) x 76 (38–408) mm. The predictive parameters for fracture were osteolytic (39% vs. 10%; p = 0.02) and permeative lesions (42% vs. 0%; p < 0.0005), a Mirels score ≥9 (42% vs. 0%; p < 0.0005), circumferential CI ≥30% (71% vs. 0%, p < 0.00001), CI ≥45 mm in height (67% vs. 0%, p < 0.00001) and CI in thickness =100% (40% vs. 0%; p = 0.0008). In the multivariate analysis, circumferential CI ≥30% was the only predictive parameter for fracture (p = 0.00035; OR = 62; CI 95%: 6.5-595). Overall survival was 91% and 40% at one month and twelve months respectively.

**Conclusions:**

Prophylactic primary fixation surgery should always be considered when the circumferential CI ≥30%.

## Introduction

The aim of extracorporeal radiotherapy of the long bones (RTLB) is to provide control of symptoms, destroy cancer cells in the treated area and prevent malignant disease-related fractures. The analgesic potential of RTLB has been demonstrated in numerous trials, with overall response rates of 60%, including 23–24% complete responses [1,2]. Bone radiotherapy is also a useful means of halting tumor proliferation and then triggering osteoblastic activity with osteoproliferation [3]. Bone recalcification after RTLB has been observed in 70% of cases, particularly in the fractionated group [3]; recalcification commenced from the first month after RTLB and peaked at three months [4]. Nonetheless, RTLB does not entirely eliminate the risk of fracture [3], particularly in the three months immediately after radiotherapy. A pathologic fracture may occur during this time. While such fractures may be atraumatic, they can also considerably aggravate morbidity and mortality.

Prophylactic surgery (followed by RTLB) should be discussed for patients with a high risk of fracture. RTLB after surgery improves bone recalcification and guarantees the stability of the new bone [5,6]. While the primary aim of fracture risk assessment is prevention, such an assessment also reduces the risk of surgical overtreatment in patients whose life expectancy is sometimes limited.

Numerous studies [1,7] have explored the risk of fracture using radiographic images (standard x-rays) to determine predictive factors. Analysis of the dimensions of metastatic lesions, and especially any cortical involvement, on standard x-rays alone remains insufficiently predictive of the fracture risk. A three-dimensional CT study provides a more precise assessment of the risk of pathologic fracture, but is not always carried out when the pain is so great that radiotherapy is urgently required. CT scan-based virtual simulation is therefore a valuable tool for providing a precise analysis of tumor infiltration and osteolysis.

The aim of our study was to use CT scan-based virtual simulation to assess the risk of fracture and identify predictive factors with a view to offering prophylactic fixation to those most at risk.

## Materials and methods

### Study design

This was a retrospective study conducted in a single center. All patients requiring analgesic radiotherapy for long bone metastasis were included. For 18 months, 47 patients were treated with RTLB (18 lung, 11 breast, 10 prostate, 8 other cancers). They had undergone a CT scan-based virtual simulation prior to radiotherapy for a long bone metastatic lesion and were enrolled between September 2010 and February 2012. Two doctors analyzed the scans before irradiation. The impending fractures were then monitored and the correlation between bone scan parameters and fracture occurrence was analyzed. All patients were seen for follow-up and treated solely at our center. Follow-up ended in June 2012: each patient had been monitored for a minimum of four months in order to screen for post-RTLB fractures. Recalcification commenced from the first month after RTLB and peaked at three months [4]; therefore, the fracture risk was considered low after the third month.

### Data collected

Using medical records, we recorded each patient's medical history, primary cancer histological type with staging, RTLB procedures, fractures and disease course. The target lesion was documented with standard x-rays, bone scintigraphy or a diagnostic CT scan.

The virtual simulation was carried out using a 16-slice GE scanner with an 80 cm ring no more than three weeks prior to the start of radiotherapy. The total dose, fractionation, X-ray energy and interval between sessions varied depending on the general condition of the patient, the intensity of pain experienced lying on the scanner bed and technical constraints.

**Figure 1:**
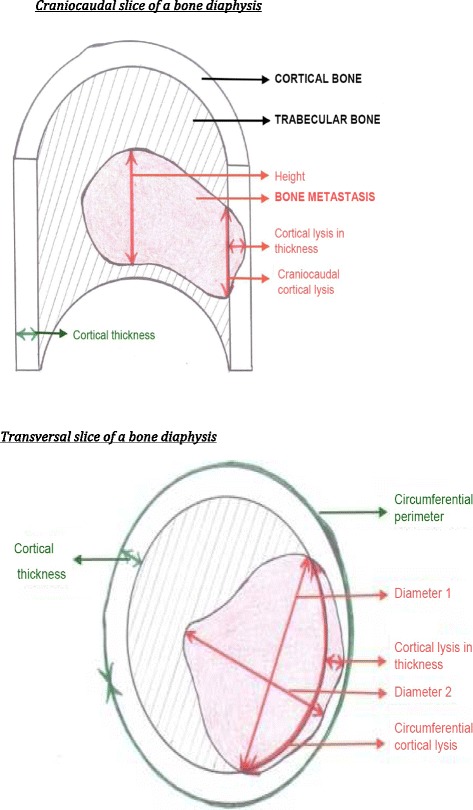
**Diagrammatic slice of a bone diaphysis.**

Two doctors (a radiotherapist and an oncologist) systematically analyzed several parameters that are known to be risk factors for pathologic fractures from previous publications [8-24]: the type and appearance of a metastasis, the mean dimensions of the lesion and the cortical involvement (CI) (craniocaudal, circumferential and thickness [Figure 1]):

- Type of metastasis: a lesion was considered to be "well defined" if its external margins were identifiable in all three spatial planes and "diffuse" if its margins were difficult to identify in the three-dimensional analysis because of an infiltrative appearance.
- Appearance of metastasis: lesions were divided into five categories. They could be "normal" (normal appearance of the bone on the scan), "osteolytic" (a primarily lytic lesion with a decrease in bone density), “osteoblastic” (a mainly blastic lesion with an increase in bone density), "mixed" (both lytic and blastic features) or "moth-eaten" (homogeneous "chewed", infiltrated appearance).
- The mean dimensions of the metastatic lesion were also measured (height, diameter in the transverse plane and CI). Lesion height (mm) was assessed by measuring the difference between the outermost transverse slice and the innermost slice on which the lesion could be seen, and then multiplying this difference by the thickness of the CT slices. "Diameter 1" (mm) was the length of the widest axis of the metastatic lesion in the transverse plane. "Diameter 2" (mm) was the largest dimension measured perpendicular to "Diameter 1" in the transverse plane. Circumference was measured in the area considered to be most at risk of fracture by both observers. Circumference perimeter was the measurement of the external perimeter of the cortical bone (mm) in the most at-risk area.
- Cortical thickness (mm) was the measurement of the thickness of the cortex considered to be normal in the most at-risk area.
- Craniocaudal cortical lysis (mm) was the measurement of the maximum CI height in the craniocaudal plane. The 30 mm threshold involvement was always recorded since this is the threshold predictive of pathological fracture according to several authors [11,22,23].
- Circumferential cortical lysis (mm) was the measurement of the diseased cortex perimeter in the most at-risk area. The circumferential lysis percentage was systematically determined by calculating the ratio of circumferential cortical lysis to the circumferential perimeter of the bone. The threshold involvement of 50% was always recorded since this is the threshold predictive of pathologic fracture according to several authors [9,10,15-17,21-23].
- Cortical thickness lysis (mm) was the measurement of the maximum thickness of cortical lysis in the at-risk area. The percentage of cortical lysis thickness was always determined.
- The Mirels score takes into account anatomical location, extent of cortical lysis, appearance of the lesion and pain intensity [21] and was calculated for each metastatic lesion. A Mirels score of nine or more was found to be predictive of fracture.
- If two lesions were present at the same time, only the lesion with the highest risk of fracture was measured.

### Statistical analysis

The statistical analysis was conducted using the *Chi*^*2*^ test and means comparison test. The performance characteristics of the craniocaudal and circumferential cortical lysis thresholds were analyzed using the *ROC curve*.

Logistic regression generalized linear modeling was used for the multivariate analysis.

The overall survival and fracture incidence curves were calculated using the Kaplan–Meier method. A *Log-Rank* test was used to compare the survival curves.

The value of p was considered significant when <0.05.

## Results

Between September 2010 and February 2012, 37 patients with 47 lesions (28 femoral, 17 humeral and two tibial) underwent analgesic radiotherapy for long bone metastasis. The patients had been monitored for a minimum of four months. The male gender ratio was 0.59 and the mean age was 62.8 years (33–93). Cancer staging was I–II for 56.8% and II–III for 43.2%. The primary cancers were lung (35.1%), prostate (27%), breast (16.3%) and others (21.6%). There were 32 adenocarcinomas, two squamous-cell cancers and three other types. At the moment their painful long bone metastasis was discovered, 18 patients had been receiving treatment with bisphosphonates and 25 with chemotherapy. Surgery was not initially performed for a variety of reasons, including poor general patient health, increase in pain refractory to medical treatment requiring urgent radiotherapy and low risk of impending fracture. Twenty-two [22] of the 47 lesions received a single dose (7 to 8 Gy). The 25 other lesions received 15 to 30 Gy in three to ten sessions over three to 19 days. The radiation dose was delivered through hard X-ray energy (5.5 to 18 MV).

**Figure 2:**
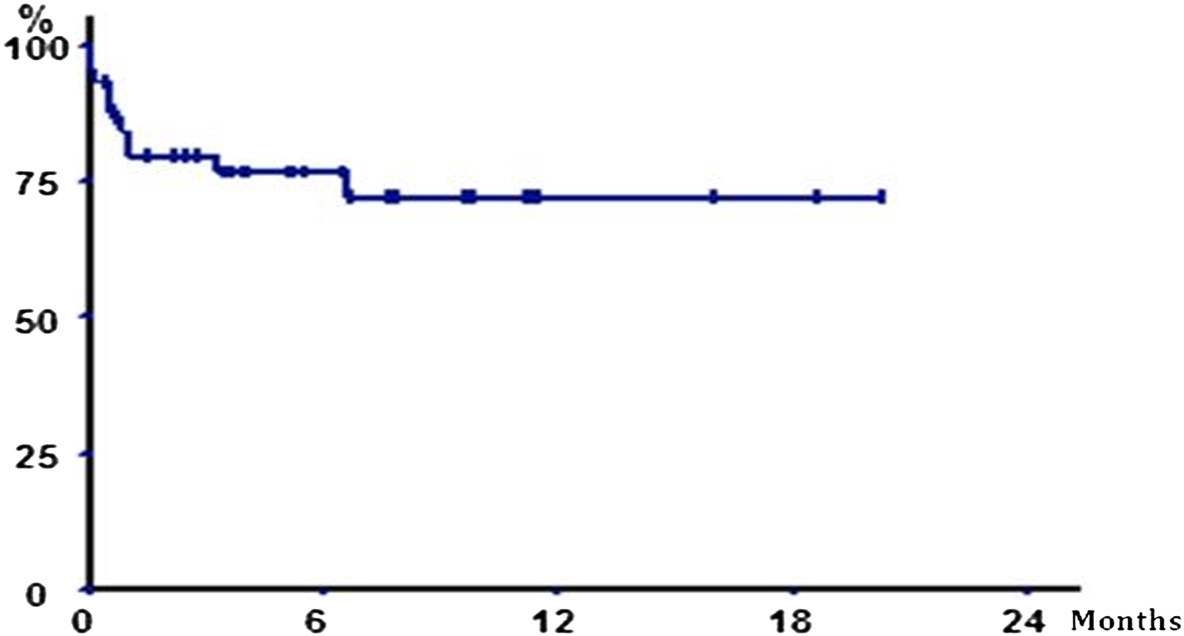
**Probability of fracture—free survival following RTLB (Kaplan—Meier).**

Ten of the 47 radiated lesions fractured during or after RTLB. When overall survival is taken into account, the incidence of fractures was 20% one month after RTLB and 25.9% at the end of the study. Two fractures occurred during RTLB, and another seven occurred in the first thirty days. The last fracture occurred at 6.6 months (Figure 2).

### Factors predictive of fracture

In the univariate analysis (Table 1), the primary cancer type, histological type, prior treatments (chemotherapy, hormone therapy, targeted therapies and radiotherapy), symptomatic treatments (bisphosphonates and corticosteroids) were not predictive of fractures. We found no statistically significant difference in anatomical lesion location (upper, middle or lower third; p = 0.85), bone type (femur, humerus, tibia), existence of local predisposing factors or radiation treatment schedule. There was no correlation between radiotherapy schedule and bone fracture: the radiotherapy procedures were the same in both groups, with or without fractures (Table 2).

**Table 1:** **Comparison of characteristics, group with fracture versus group without fracture (Chi 2 test, comparison of means) a) Clinical parameters**

	**Total (%)**	**Fractures (%)**	**Without fractures (%)**	
**Number of patients**	37	10	27	
**Number of lesions radiated**	47	10	37	
**Age**				p = 0.16
Mean (years)	62.8	65.3	62.2	
Age range (years)	33 - 93	33 - 87	33 - 93	
**Gender**				NS
Men	27 (57.5)	4 (14.8)	23 (85.2)	
Women	20 (42.5)	6 (30)	14 (70)	
**Primary cancer**				NS
Lung	18 (38.3)	4 (22)	14 (78)	
Breast	11 (23.4)	3 (27)	8 (73)	
Prostate	10 (21.3)	---	10 (100)	p = 0.08
Other	8 (17.0)	3 (37.5)	5 (62.5)	
**Corticosteroids**				NS
< 1 month	1 (2.1)	1 (100)	---	
> 1 month and < 6 months	2 (4.2)	1 (50)	1 (50)	
> 6 months	0	---	---	
No	42 (89.5)	8 (19)	34 (81)	
NA	2 (4.2)	---	2 (100)	
**Biphosphonates**				NS
Yes	21 (44.7)	4 (19)	17 (81)	
No	21 (44.7)	4 (19)	17 (81)	
NA	5 (10.6)	2 (40)	3 (60)	
**Chemotherapy ongoing**				p = 0.03
Yes	34 (72.3)	10 (29)	24 (71)	
No	13 (27.7)	---	13 (100)	
**Long bone**				NS
Femur	28 (59.6)	7 (25)	21(75)	
Humerus	17 (36.2)	3 (18)	14(78)	
Tibia	2 (4.2)	---	2 (100)	
**Locoregional history**				NS
Fracture	2 (4.2)	1 (50)	1 (50)	
Osteoarthritis	5 (10.5)	---	5 (100)	
No	40 (85.1)	9 (23)	31 (77)	
**Contralateral prosthesis**	4 (8.5)	---	4 (100)	NS
**Pain intensification**				NS
Yes	42 (89.4)	10 (24)	32 (76)	
No	5 (10.6)	---	5 (100)	
***a) Radiological parameters***				
**Number of lesions radiated**	47	10	37	
**Localization**				NS
Upper third	43 (91.5)	9 (21)	34 (79)	
Lower third	4 (8.5)	1 (25)	3 (75)	
**Type of metastatic spread**				
Well defined	23 (49.0)	---	23 (100)	p = 0.0005
Diffuse	24 (51.0)	10 (42)	14 (58)	
**Appearance of the metastatic lesion**				
Normal	2 (4.2)	---	2 (100)	p = 0.02
Osteolytic	18 (38.3)	7 (39)	11 (61)	
Mixed	15 (31.9)	3 (20)	12 (80)	
Osteoblastic	11 (23.5)	---	11 (100)	
Moth---eaten	1 (2.1)	---	1 (100)	
**Mean dimensions (mm)**				
Height	59.8	80.07	46.1	
CI 95%	(40.5; 79.1)	(50.37; 109.8)	(33.3; 58.9)	p < 0.01
Diameter 1	32.2	48.5	27.6	
CI 95%	(26.8; 37.5)	(41.8; 55.1)	(22.7; 32.6)	p = 0.01
Diameter 2	22.9	34.2	19.8	
CI 95%	(18.6; 27.3)	(29.5; 38.8)	(15.3; 24.3)	p = 0.01
Circumferential perimeter	142.6	133.1	145.2	p = 0.15
CI 95%	(129.9; 155.3)	(127.1; 139.1)	(130.3; 160.1)	
Cortical thickness	3.5	3.3	3.6	p = 0.27
CI 95%	(3.1; 3.9)	(3.2; 3.4)	(3.1; 4.0)	
**Craniocaudal cortical lysis**				p < 0.0001
Mean (mm)	45.2	103.0	28.6	
CI 95%	(25.3; 65.0)	(85.6; 120.4)	(14.7; 42.6)	
Cortical lysis threshold				
No	16(34.0)	---	16 (100)	
< 30 mm	10 (21.3)	---	10 (100)	
≥ 30 mm	21 (44.7)	10 (47.6)	11 (52.4)	
**Circumferential cortical lysis**				p < 0.0001
Mean (mm)	32.3	78.6	19.8	
CI 95%	(22.3; 42.3)	(74.5; 82.6)	(11.6; 28.1)	
Cortical lysis (%)				
No	16 (34.0)	---	16 (100)	
< 50%	21 (44.7)	2 (9.5)	19 (90.5)	
≥ 50%	10 (21.3)	8 (80)	2 (20)	
**Cortical lysis in thickness**				p = 0.0018
Mean (mm)	2.2	3.3	2.0	
CI 95%	(1.6; 2.9)	(3.2; 3.4)	(1.2; 2.7)	
Cortical lysis (%)				
No	16 (34.0)	---	16 (100)	
0 – 99%	5 (10.7)	---	5 (100)	
100%	26 (55.3)	10 (38.5)	16 (61.5)	
**Mirels score**				P = 0.0005
≤ 7	14 (29.8)	---	14 (100)	
= 8	9 (19.1)	---	9 (100)	
≥ 9	24 (51.1)	10 (42)	14 (58)	

CI: confidence Interval; NA: not applicable; NS: not significant.

**Table 2:** **Comparison of fractionation type, group with fracture versus group without fracture**

	**N (%)**	**Fractures (%)**	**Without fractures (%)**
**Number of lesions radiated**	47	10	37
**Dose received**			
**Single fraction**	22 (46.8)	5 (22.7)	17 (77.3)
7 Gy	3 (6.4)	---	3 (100)
8 Gy	19 (40.4)	5 (26.3)	14 (73.7)
**Multiple fractions**	25 (53.2)	5 (20)	20 (80)
15 Gy	1 (2.1)	---	1 (100)
20 Gy	13 (27.7)	3 (23.1)	10 (76.9)
25 Gy	3 (6.4)	---	3 (100)
30 Gy	8 (17.0)	2 (25)	6 (75)

The risk factors for impending fracture were as follows: an osteolytic (39% vs. 10%; p = 0.02) and diffuse appearance (42% vs. 0%; p < 0.0005), circumferential CI ≥50% (80% vs. 5%, p < 0.00001) and ≥30% (71% vs. 0%, p < 0.00001), height of involvement ≥30 mm (48% vs. 0%, p < 0.00001) and ≥45 mm (67% vs. 0%, p < 0.00001) and cortical thickness =100% (38% vs. 0%; p = 0.0008). A Mirels score ≥9 (42% vs. 0%; p < 0.0005) was also predictive of fracture.

**Figure 3:**
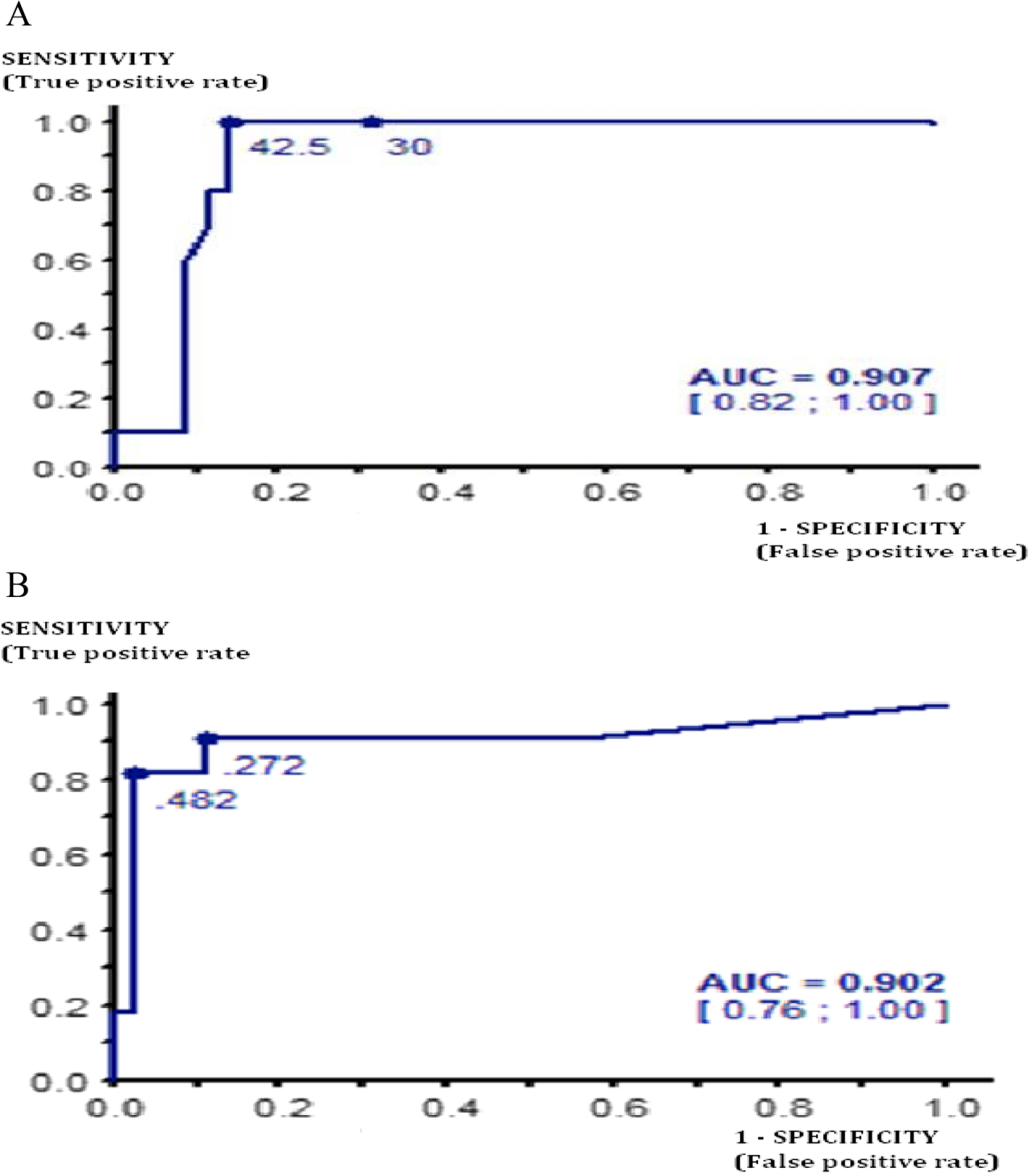
**ROC curve. A)** Performance of the craniocaudal cortical lysis threshold. No risk of fracture if the craniocaudal cortical involvement is less than 42.5 mm. 45 mm threshold: sensitivity = 100%, specificity = 85.7%, positive predictive value = 66.7%, negative predictive value = 100%. **B)** Performance of the circumferential cortical involvement threshold. No risk of fracture if the circumferential cortical involvement is less than 27%. 30% threshold: sensitivity = 100%, specificity = 89%, positive predictive value = 71%, negative predictive value = 100%.

The craniocaudal and circumferential involvement thresholds with the greatest sensitivity and specificity in the study population were ≥45 mm for craniocaudal involvement and ≥30% for circumferential involvement (Figure 3).

In the multivariate analysis, only circumferential involvement ≥30% was predictive of fracture (p =0.00035; OR =62; CI 95% = [6.45 – 595]).

### Overall survival

The mean follow-up was 5.95 months (0.43–20.27 months) for the 37-patient study population. No patients were lost to follow-up.

**Figure 4:**
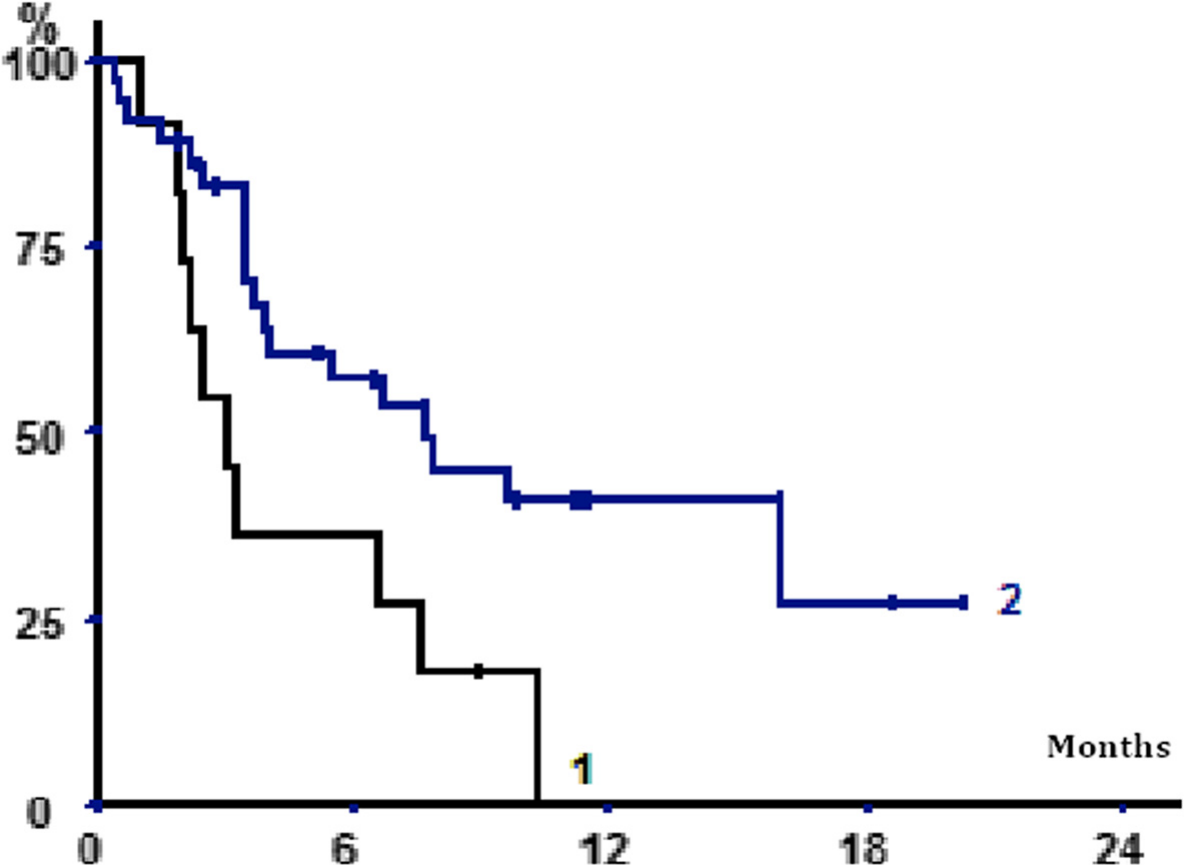
**Overall survival after RTLB as a function of fracture incidence.** Curve 1: with fracture. Curve 2: without fracture Overall survival curve, Kaplan–Meier; Log--‐Rank, p = 0.014.

Overall survival was 91%, 55% and 40% at one month, six months and one year respectively (Figure 4). Overall survival was significantly lower in the patients presenting with fractures (p = 0.014).

## Discussion

Our study showed that more than 25% of patients undergoing radiotherapy for metastases presented with a fracture. In the multivariate analysis, the only factor predictive of fractures identified during the CT-scan-based virtual simulation study was circumferential a CI ≥30%.

Several studies [1,7] have explored risk factors for fractures using radiographic images (standard X-rays) to determine predictive factors. In general, high impending fracture risk lesions are lytic [8-17], proximal [10,18,19], large (>25 mm) [8-10] and femoral (Harrington criteria, [10,25]). They trigger increasing pain [8-11,13-15,17,20,21], involve more than 50% of the cortex circumference [9,10,15-17,21-23] with craniocaudal cortex involvement of over 30 mm [11,22-24] and a Mirels score of ≥9 [21].

As in other studies, we found that the other radiological parameters predictive of fracture were a lytic, diffuse, poorly circumscribed appearance and cortical involvement (30% circumferential, 45 mm height and 100% thickness). In our study, ≥30 mm femoral craniocaudal cortical involvement was a significant predictor of fracture risk in the univariate analysis, but this consideration could have led to 14 unnecessary surgical procedures (37.8% false positive rate).

In our series, a composite Mirels score of ≥9 was also predictive of fracture. However, while this score has the advantage of being very sensitive, it lacks specificity [7,23,24]. Therefore, in our study this score was ≥9 in 100% of the fracture cases, but there were 14 false positives (37.8%). The three-dimensional study clearly provides a more precise assessment of the risk of pathologic fracture. Measuring circumferential cortical involvement enhances this assessment: primary surgical fixation should be considered in patients with circumferential cortical involvement ≥30%.

Our study was representative and comparable with those reported in other series in the literature in terms of age, gender, primary cancer type, performance status and fracture rates [6,15,24,26-28]. In our series, radiotherapy procedures and fractionation did not affect the fracture incidence and the data in the literature are discordant on this point [26,28]. Furthermore, in our population, bisphosphonate administration did not influence the risk of bone fractures.

Nevertheless, our study has several limitations:

- It is retrospective in design and our population was very small. However, all the patients treated with RTLB were enrolled and none were lost to follow-up, which represents a real-life experience.
- The four-month follow-up period could also be seen as a limitation of our study. However, 90% of the fractures occurred in the month following RTLB and it has been shown that bone recalcification is obtained three months after RTLB, after which time the risk of fractures is very low [4].
- While our scan images were read by a radiotherapist and an oncologist rather than a radiologist, these are the healthcare professionals who are required to assess fracture risk in patients with painful bone metastases on a daily basis. Our patients were oriented directly for urgent analgesic radiation and a CT scan was performed promptly for the virtual simulation (without evaluation by a radiologist). At the same time, we recorded bone scan parameters for this study.
- Due to the retrospective nature of the study, some risk factors for fracture could not be included, such as assist devices, weight-bearing status, bone density status, smoking status and osteoporosis comorbidity.

Our data must be confirmed in a prospective study including a much larger series of patients, since it is important to precisely establish when prophylactic fixation is required to reduce morbidity and mortality [5,6,10,15]. The development of an instrument that identifies patients who have a relatively high risk of developing such a fracture and therefore should be considered candidates for surgical stabilization is helpful. This strategy could optimize the management of fragile metastatic patients. Elective surgery in patients in good general health is simpler and less risky than an emergency procedure, with more rapid relief of pain and recovery of mobility [29,30]. Surgical overtreatment also unnecessarily increases morbidity (e.g., hospitalization, general anesthetics and complications arising from a forced supine position) in patients whose life expectancy is limited. The appropriate management of palliative patients and cost-effective proactive approaches may offer more clinical benefit and value for carefully selected patients.

## Conclusions

This study analyzes the risk of fractures following radiation for bone metastasis and attempts to determine which patient population would benefit from prophylactic surgery. Circumferential cortical involvement is easy to measure and should be systematic during CT scan-based virtual simulation prior to radiotherapy.

### Consent

Written informed consent was obtained from the patient for the publication of this report and any accompanying images.
